# Mycobacterium szulgai Pulmonary Infection in an Immunocompromised Patient

**DOI:** 10.7759/cureus.51388

**Published:** 2023-12-31

**Authors:** Sara Neves, Ema Pos, Ana Horta, António Ludgero Vasconcelos

**Affiliations:** 1 Infectious Diseases Department, Centro Hospitalar Universitário de Santo António, Porto, PRT

**Keywords:** crohn`s disease, tumor necrosis factor-alpha (tnf-α) inhibitors, mycobacterium szulgai, nontuberculous mycobacteria (ntm), adalimumab

## Abstract

*Mycobacterium* *szulgai* is a slow-growing nontuberculous mycobacterium (NTM). It was first described in 1972 and is responsible for less than 0.2% of all NTM infections. The most common presentation resembles pulmonary tuberculosis, but it may also present as an extrapulmonary disease. It primarily affects individuals with underlying lung disease or immunocompromising conditions. The increasing use of tumor necrosis factor-alpha inhibitors, such as adalimumab, is associated with an increased risk of serious infections. We report a case of *Mycobacterium szulgai* infection in a 23-year-old woman with a history of childhood pneumonia and Crohn’s disease on adalimumab.

## Introduction

*Mycobacterium szulgai* (*M. szulgai*) is a slow-growing nontuberculous mycobacterium (NTM), first described in 1972. Despite rarely causing infection in humans (less than 0.2% of all NTM infections), its isolation in culture is often clinically significant. The most common presentation resembles pulmonary tuberculosis, but it may also present as an extrapulmonary disease. Infection primarily occurs in individuals with underlying lung disease or immunocompromising conditions [1-4].

According to the American Thoracic Society/Infectious Diseases Society of America (ATS/IDSA) clinical practice guideline, the diagnosis of NTM pulmonary disease is confirmed when clinical, radiological, and microbiological criteria are present. Regarding the latter, if sputum culture is used, a minimum of two positive samples is required to rule out sample contamination [5].

Tumor necrosis factor-alpha (TNF-α) inhibitors, such as adalimumab, are an important therapeutic tool in the treatment of various inflammatory conditions, including rheumatoid arthritis, inflammatory bowel disease, and psoriasis, and their use is gradually becoming more widespread. However, TNF-α inhibitors target one of the main pathways responsible for the immune response to Mycobacteria, which can lead to a consequent rise in the number of infections caused by NTM [6].

We describe a case of pulmonary infection with *M. szulgai* in a young woman with Crohn’s disease treated with adalimumab. To our knowledge, this is the second report of *M. szulgai* infection in Portugal [7].

## Case presentation

We present the case of a 23-year-old woman with a history of childhood pneumonia and Crohn's disease, on adalimumab for 10 months. She was followed up in the Infection Prevention in Immunocompromised Patients outpatient clinic. During a routine appointment, when asked about any new symptoms since the last visit, she reported a six-month history of productive cough without accompanying symptoms, specifically fever, night sweats, or weight loss. Her primary care physician had seen her a month earlier and ordered a chest X-ray, which revealed an opacity in the right paracardiac region consistent with pneumonia. She was prescribed a three-day course of azithromycin, but the symptoms persisted.

**Figure 1:**
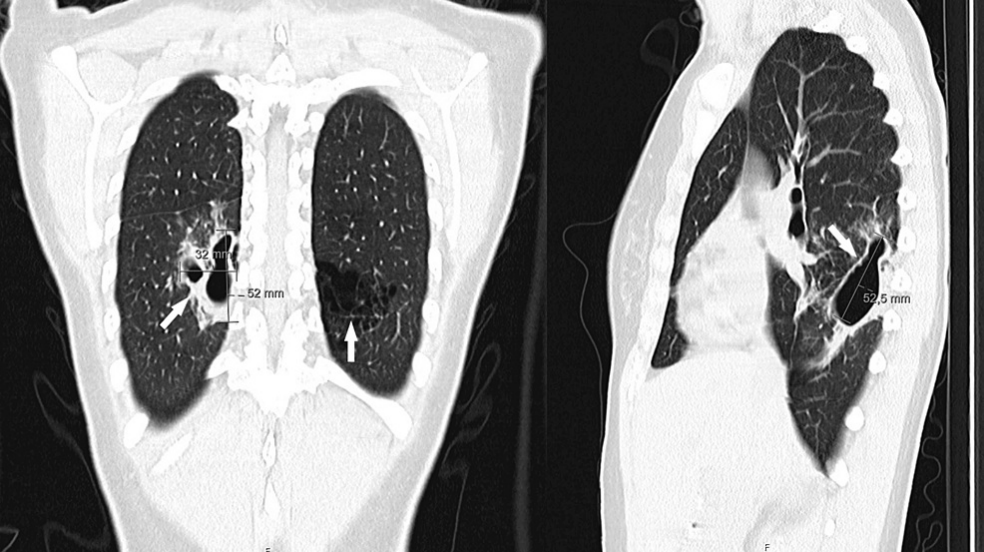
Chest CT scan showing two adjacent cavitations with thick walls on the right lower lobe

The patient had recently been screened for latent tuberculosis using a Mantoux tuberculin skin test and an interferon-gamma release assay (IGRA), which were negative. Chest computed tomography (CT) showed two adjacent cavitations in the right lower lobe with thick walls (44x32x52 mm), contiguous atelectasis, and several diffuse centrilobular micronodules, suggestive of pulmonary tuberculosis; and lower left lobe bronchiectasis consistent with bronchiolitis obliterans's sequelae (Figure 1).

At this point, adalimumab treatment was immediately discontinued. Polymerase chain reaction (PCR) for *Mycobacterium tuberculosis *and auramine staining were negative in two subsequent sputum samples, but mycobacterial culture showed growth of *Mycobacterium *spp. Samples were sent to the national reference laboratory, which identified the isolate as *Mycobacterium szulgai*. Bronchoscopy was also performed, and bronchoalveolar lavage (BAL) culture confirmed the diagnosis.

**Figure 2:**
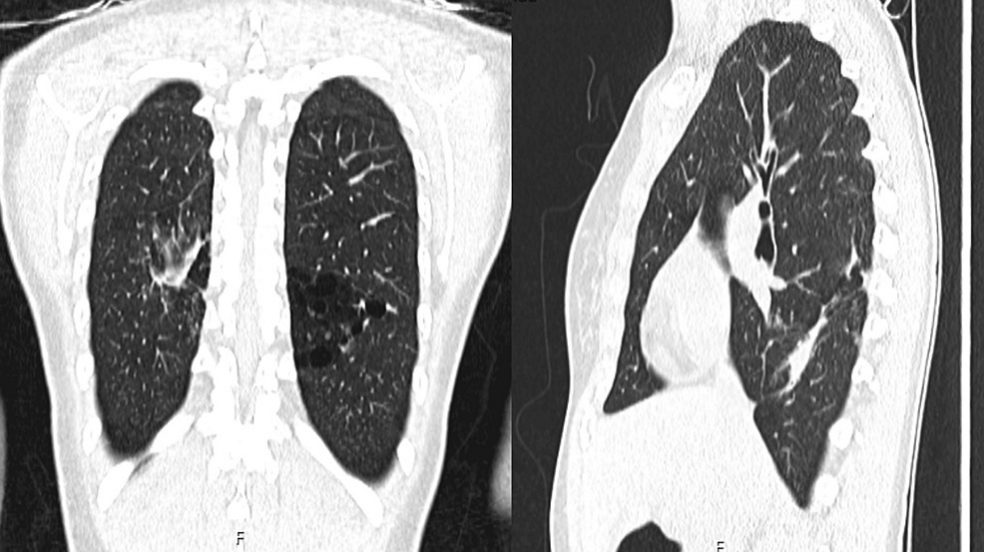
Follow-up chest CT scan three months after treatment completion

The patient was referred to the local Pneumological Diagnostic Center, part of a national network of outpatient clinics specialized in the diagnosis and treatment of diseases caused by mycobacteria, where she started treatment with isoniazid (300 mg once a day), rifampin (600 mg once a day), and ethambutol (15 mg/kg once a day). Drug susceptibility testing (DST) for *Mycobacterium szulgai* is unavailable in Portugal, but clinical improvement and culture conversion supported treatment efficacy. The patient completed 16 months of treatment counting from culture conversion with complete resolution of symptoms and radiological response (Figure 2).

## Discussion

This case concerns a young woman with pulmonary sequelae of bronchiolitis obliterans, treated with a TNF-α inhibitor for Crohn's disease, conditions which are recognized as predisposing to NTM infection [1-2]. She presented with a productive cough persisting for several months, and cavitations, bronchiectasis, and diffuse centrilobular micronodules on a chest CT scan, suggestive of pulmonary tuberculosis.

The patient exhibited clinical criteria (respiratory symptoms), radiological findings (as described above), and microbiological evidence (isolation in culture from two sputum samples and BAL) consistent with pulmonary infection by *M. szulgai*, in accordance with the ATS/IDSA clinical practice guidelines.

There are no treatment recommendations for *M. szulgai* infection based on randomized clinical trial data. According to the available literature, *M. szulgai* is often resistant to isoniazid and sensitive to clarithromycin and rifampicin *in vitro*. Based on the limited evidence available, multidrug therapy is recommended, including at least three drugs selected according to DST, usually a combination of a macrolide (azithromycin or clarithromycin), rifampin, and ethambutol. Regarding treatment duration, guidelines recommend 12 months of therapy in total or counting from culture conversion when a macrolide, rifampin, or ethambutol cannot be used [8].

In this case, the chosen regimen (with isoniazid instead of a macrolide) was decided by the physician at the Pneumological Diagnostic Center. The reasons for this choice could not be discussed. The decision was not guided by DST due to its unavailability. The patient completed 16 months of treatment counting from culture conversion. It is worth noting that eight patients with *M. szulgai* infection, treated with a similar regimen for a shorter duration, were included in a South Korean study where no recurrence was reported [9].

With the increasing prevalence of autoimmune and neoplastic diseases and the expanding use of therapeutic strategies that induce varying degrees of immunosuppression, there has been a growing need to establish clinics with the goal of preventing infections through immunization, chemoprophylaxis, education, and awareness of symptoms; early diagnosis through screenings, symptom inquiries, or other complementary tests; and treatment or referral to appropriate care. In the Infection Prevention in Immunocompromised Patients outpatient clinic, patients are ideally seen before starting immunosuppression and are followed annually or according to the periodicity dictated by their underlying condition.

In this case, the patient sought medical attention from her primary care physician several months after the onset of symptoms and awaited her scheduled appointment for specialist evaluation. Despite not progressing to a more severe disease, cases of disseminated infection by this microorganism have been described, emphasizing the importance of timely diagnosis and treatment.

## Conclusions

To our knowledge, this is the second reported case in Portugal of *Mycobacterium szulgai* infection, aligning with the evidence that this microorganism rarely causes infection in humans. However, the increasing use of immunosuppressive drugs such as TNF-α inhibitors, which carry an elevated risk of serious infections, is likely to be accompanied by a consequent rise in the number of infections caused by NTM. With this report, we aim to draw attention to this diagnosis and emphasize the importance of educating patients on the recognition of symptoms consistent with these conditions. It is in the Infection Prevention in Immunocompromised Patients clinic that we have the opportunity to do so, a field of Infectious Diseases that has proven crucial in the prevention, early diagnosis, and treatment of infections.
